# Core temperature-dependent leukocyte and neutrophil responses during prolonged heat exposure

**DOI:** 10.3389/fimmu.2026.1798608

**Published:** 2026-04-22

**Authors:** Yi Xu, Haojian Wang, Fèlix Faming Wang

**Affiliations:** Centre for Molecular Biosciences and Non-Communicable Diseases Research, College of Safety Science and Engineering, Xi’an University of Science and Technology, Xi’an, China

**Keywords:** acute inflammation, core temperature, immune cell counts, prolonged heat exposure, sex differences

## Abstract

**Background:**

Prolonged indoor heat exposure disrupts immune homeostasis and can precipitate acute systemic inflammation. However, the core temperature threshold triggering sex-specific immune cell (leukocytes and neutrophils) mobilization during passive indoor heat stress remains undefined.

**Methods:**

We studied 68 males and 46 females exposed to wet-bulb temperatures (*T*_w_) of 32–35 °C. Rectal temperature (*T*_rec_) was continuously monitored, and blood samples were collected at 0.5 °C increments up to 38.9 °C. Leukocyte and neutrophil counts were modeled using quadratic and segmented mixed-effects models to identify inflection points of immune activation.

**Results:**

Both leukocytes and neutrophils increased nonlinearly with rising *T*_rec_ (*p* < 0.05). Estimated *T*_rec_ breakpoints occurred at approximately 38 °C, with overlapping 95% confidence intervals across sexes and cell types, indicating a transition to more rapid immune cell mobilization. This breakpoint was comparable to commonly cited thresholds for limiting excessive heat strain. Below the breakpoint, females exhibited steeper increases in both leukocytes and neutrophils, whereas above it, males showed greater acceleration, particularly for leukocytes. Neutrophil responses were consistently greater in males across the full temperature range (36.4–38.9 °C).

**Conclusions:**

A distinct core temperature threshold (~ 38.0 °C) governs accelerated immune cell mobilization and reveals sex-dependent response patterns. These findings provide an immunological rationale for current occupational heat limits and emphasize the importance of integrating sex-specific considerations into protective guidelines under extreme heat conditions.

## 1 Introduction

Global warming has intensified the frequency and duration of extreme heatwaves, exposing millions to conditions that can destabilize immune and thermoregulatory homeostasis (1–3). Even in healthy adults, sustained thermal stress can provoke systemic inflammation through activation of innate immune pathways (4). Leukocytes, particularly neutrophils, play a central role in initiating and amplifying inflammatory responses to thermal stress (5, 6). Yet the relationship between rising core body temperature and immune cell mobilization under prolonged passive heat exposure—without the confounding effects of exercise—remains poorly characterized.

Rectal temperature (*T*_rec_), a commonly used surrogate for pulmonary artery temperature (7), is widely employed to assess internal heat strain. As wet-bulb temperature (*T*_w_), which integrates ambient temperature and humidity and reflects the capacity for evaporative heat loss, increases, evaporative heat dissipation becomes progressively constrained, and *T*_rec_ rises even at rest during prolonged exposure (8, 9). Recent evidence suggests that thermoregulatory limits during prolonged passive heat exposure may occur at lower *T*_w_ values than the widely cited theoretical threshold of 35 °C, highlighting the importance of understanding how increasing internal heat strain relates to physiological responses under sustained heat stress (9, 10). Current occupational and public health guidelines designate 38.0 °C as the upper safe limit for core temperature (11–13), a threshold historically linked to maximal sustainable metabolic workloads (~ 490 W) rather than to immune or inflammatory endpoints. Consequently, the immunological validity of this widely adopted threshold remains uncertain.

Sex-based differences in thermoregulation and immune function are well established (14, 15). Estrogen and testosterone modulate inflammatory signaling in opposing directions: females often show earlier immune activation and enhanced anti-inflammatory control, whereas males possess larger bone marrow reserves and can mount delayed but stronger responses (16, 17). These differences suggest that sex-specific inflection points in immune mobilization may emerge as core temperature rises, yet no prior work has quantified these relationships under controlled, prolonged, passive heat exposure.

Mechanistically, heat-induced leukocyte mobilization is thought to involve coordinated actions between cytokines and chemokines that regulate immune cell production and trafficking. Granulocyte colony-stimulating factor (G-CSF) promotes neutrophil proliferation and mobilization from bone marrow, while chemokines such as interleukin-8 (IL-8/CXCL8) and C-X-C motif chemokine ligand 1 (CXCL1) contribute to neutrophil recruitment to peripheral tissues (18, 19). In parallel, sympathetic activation and hypothalamic–pituitary–adrenal (HPA) signaling also modulate immune cell redistribution and priming (20). These neuroendocrine responses may contribute to the mobilization of circulating leukocytes and neutrophils observed during heat stress (21, 22), which serve as accessible indicators of early systemic stress response during heat exposure and may represent components of the broader physiological cascade associated with severe heat strain. Identifying the core temperature at which these cytokine–chemokine–neuroendocrine interactions begin to accelerate may provide a physiological basis for interpreting current occupational heat exposure limits.

Leukocyte and neutrophil counts are widely used to assess immune cell mobilization during stress conditions, reflecting recruitment into circulation. Nonetheless, these counts do not directly measure immune cell activity, maturity, or functional capacity, such as phagocytosis, oxidative burst, or chemotaxis. While these markers provide valuable insights into immune cell dynamics, we acknowledge that they cannot offer a complete picture of immune function. This study, therefore, serves as a first step in understanding the broader physiological effects of prolonged indoor heat exposure.

We systematically exposed healthy young adults to *T_w_* = 32–35 °C under 12 combinations of temperature and humidity, continuously monitoring *T_rec_* and sampling blood at 0.5 °C increments up to 38.9 °C. The present analysis was derived from this broader experimental protocol; however, to avoid statistical dependence arising from repeated participation across conditions, one trial was randomly selected from each participant for inclusion in the final analytical dataset. This design ensured that physiological and immunological responses were evaluated as a function of internal heat strain (*T*_rec_), rather than elapsed exposure time. Because the experiments were conducted in a controlled indoor climate chamber with minimal radiant heat sources, *T*_w_ was selected as the primary environmental index to characterize the heat stress imposed on participants. Under these conditions, evaporative limitation was the dominant external constraint on body heat dissipation, and *T*_w_ —integrating ambient temperature and humidity — provides a physiologically relevant metric linking environmental heat exposure to progressive increases in core temperature. We hypothesized that leukocyte and neutrophil counts would increase in a nonlinear, temperature-dependent manner, with sex-specific differences in threshold and rate of activation. By mapping immune cell dynamics across the full thermal range, this study aims to identify the critical core temperature inflection point that marks the onset of systemic immune mobilization and to evaluate its alignment with existing occupational heat exposure limits.

## 2 Methods

### 2.1 Participants, exposure conditions and ethical approval

Experiments were conducted in a climate chamber (SEWTH-A-290H, Espec Corporation, Osaka, Japan; temperature accuracy: ± 0.5 °C; humidity deviation: -3% to +2% RH) under four wet-bulb temperature (*T*_w_) conditions: 32 °C, 33 °C, 34 °C, and 35 °C. Each *T*_w_ condition was achieved using three different combinations of dry-bulb temperature (*T*_db_) and relative humidity (RH), calculated according to the Stull equation (23). A total of 68 males (age: 24.5 ± 2.4 yr; body mass index [BMI]: 22.1 ± 1.7 kg/m²) and 58 females (age: 23.2 ± 2.0 yr; BMI: 20.8 ± 2.1 kg/m²) completed the heat exposure trials. Of these, 20 males and 16 females completed all trials across all four *T*_w_ conditions, whereas an additional 48 males and 42 females completed only the *T*_w_=32 °C trials conducted at a dry-bulb temperature (*T*_db_) of 40 °C. Trials were conducted between March 2023 and May 2025, excluding the summer months (June–September) to minimize potential effects of seasonal heat acclimatization. The order of trials was randomized, with a washout period of approximately one week between experimental sessions to reduce carryover effects. Detailed anthropometric characteristics for each experimental condition are provided in **Supplementary Table 1**. Female participants reported regular menstrual cycles and were tested during the follicular phase (4 ± 3 days after the onset of menstruation) to minimize hormonal variability (24, 25). All participants underwent medical screening at a certified hospital to exclude cardiovascular, endocrine, or immune disorders. In addition, none had engaged in occupational heat exposure or heat acclimatization training within the preceding six months.

Ethical approval was obtained from the Institutional Review Board (IRB) of Xi’an University of Science and Technology (approval nos. XUST-IRB223011 and XUST-IRB224002). All participants provided written and verbal informed consent before enrollment, in accordance with the Declaration of Helsinki, and received financial compensation for their time. Prospective registration in a public clinical trial registry was not required, as the study did not involve evaluation of a clinical or therapeutic intervention.

### 2.2 Experimental procedures

Participants were instructed to drink ≥ 1000 mL of water during the 24 h preceding each trial and to refrain from caffeine, alcohol, tea, spicy foods, and medications. Trials began at 09:00, with participants instructed to wear standardized clothing ensembles, including short-sleeve T-shirts, underwear, trousers, socks, and sports shoes; females additionally wore sports bras, yielding insulation values of 0.40 clo for males and 0.41 clo for females. Prior to entering the environmental chamber, eligibility criteria were confirmed, including urine specific gravity (USG) < 1.020 and rectal temperature (*T*_rec_) ≤ 37.1 °C, measured using a digital refractometer (PAL-10S, ATAGO Co. Ltd., Tokyo, Japan) and a rectal thermistor probe (YSI401, Yellow Spring Instrument, Yellow Springs, OH, USA, accuracy: ± 0.1 °C), respectively. Pre-exposure *T*_rec_ was defined as baseline.

During heat exposure, participants remained seated and performed light office work (reading or computer tasks). To maintain euhydration, participants were provided with a warm electrolyte solution (~ 37 °C) at a prescribed rate of 8–10 mL·kg^-^¹·h^-^¹ and were permitted to consume additional fluids *ad libitum*. Actual fluid intake was recorded throughout the experiment, and hydration-related variables are reported in **Supplementary Tables 1** and **2**. Heart rate and blood pressure were monitored throughout the exposure, and nude body mass was measured before and after exposure to assess hydration status. Fingertip blood samples were collected at predefined intervals for leukocyte and neutrophil counts, as detailed in Section 2.3. After 3 hours of exposure, participants received a standardized 550-kCal sandwich meal. Restroom breaks were permitted in a thermoneutral room (23–27 °C, 40–60% RH), averaging 2.6 ± 1.2 visits per exposure, each lasting < 3 min.

The experiment was terminated if any of the following criteria were met: 1) rectal temperature (*T*_rec_) reached 39.0 °C; 2) sustained cardiovascular strain, defined as a heart rate exceeding 90% of the age-predicted maximum (HR_max_ = 208 − 0.7 × age) for more than 5 consecutive minutes (26), or systolic blood pressure below 90 mmHg (27); 3) onset of severe subjective symptoms (e.g., uncontrollable hyperventilation, early heat-induced tetany, dizziness, limb numbness, nausea, pronounced discomfort, and altered mental state); or 4) the supervising nurse judged continued exposure to be unsafe. Further details are available in the previously established protocol (9).

### 2.3 Blood sample collection

Capillary (fingertip) blood samples were obtained for leukocyte and neutrophil counts, as prior studies have demonstrated strong agreement with venous measurements and no clinically meaningful differences in adults (28, 29). Before heat exposure, participants were seated for 30 min prior to baseline sampling. Fingertip blood (~60 µL) was collected using a single-use 28G lancet (Sinocare, Changsha, China) following disinfection with 75% medical alcohol and complete air-drying. The first drop of blood was discarded to reduce contamination from tissue/interstitial fluids and minimize hemolysis. The pre-exposure blood sample was defined as baseline. During heat exposure, additional samples were collected whenever *T*_rec_ increased by 0.5 °C. Each sample was immediately mixed with EDTA-K_2_ anticoagulant and analyzed on-site using an automated hematology analyzer validated for small-volume capillary specimens (BHA-3000, Getein, Nanjing, China). All samples were measured in triplicate and averaged, with routine internal quality control performed throughout data collection.

Data quality control procedures were applied prior to analysis. First, for breakpoint estimation, each participant’s trial dataset was required to include at least three blood samples to ensure sufficient data points for piecewise modeling; datasets with fewer than three samples were excluded from the final analysis. Second, participants with abnormal baseline leukocyte (< 3.5×10^9^/L or > 9.5×10^9^/L) or neutrophil counts (< 1.8×10^9^/L or > 6.3×10^9^/L) were excluded during baseline screening to reduce potential confounding from underlying inflammatory or hematological conditions, based on established hematology reference intervals (30). Third, to ensure adequate representation of the upper temperature range, each participant was required to have at least one blood sample collected at *T*_rec_ > 38.0 °C. Fourth, when participants completed multiple experimental conditions, one condition was randomly selected for analysis so that each participant contributed data from only one trial, minimizing statistical dependence arising from repeated participation. Following these criteria, a total of 68 males and 46 females were included in the final analysis, yielding 294 measurements in males and 179 measurements in females.

### 2.4 Data analysis

All statistical analyses and figures were conducted in R and RStudio (version 4.5.0). First, a generalized additive mixed model (GAMM; *mgcv* package) was used to characterize the relationship between rectal temperature (*T*_rec_) and immune cell counts without prespecifying its functional form. The model included a penalized spline for *T*_rec_, experiment condition as a fixed effect, and participant ID as a random intercept, and was fitted using penalized restricted maximum likelihood (REML). Effective degrees of freedom (edf) > 1 and *p* < 0.05 indicated a significant nonlinear trend.

Guided by the nonlinear patterns observed, parametric fixed-effects models (linear, polynomial, and power regression) were then fitted to the log-transformed immune cell counts. Sex-stratified piecewise linear mixed-effects models (*lme4* package) were applied to identify *T*_rec_ breakpoints at which the rate of change in cell counts significantly increased. Breakpoints were estimated using piecewise linear mixed-effects models fitted to the full analytical dataset, rather than being estimated separately for each participant and subsequently aggregated. Breakpoint locations were determined through an iterative grid search across a physiologically plausible range of rectal temperatures (*T*_rec_; 36.4–39.0 °C, in increments of 0.05 °C). The models included experimental condition as a fixed effect and participant ID as a random intercept to account for repeated measures, and were estimated using maximum likelihood (ML). As a sensitivity analysis, elapsed time from baseline to each blood sampling point was additionally included as a fixed effect in the piecewise mixed-effects models to evaluate whether the estimated *T*_rec_ breakpoint was influenced by exposure duration. The presence of a breakpoint was evaluated via likelihood ratio tests comparing piecewise and linear mixed-effects models, with Bonferroni adjustment applied for four comparisons. Model diagnostics were performed through visual inspection of residual plots, including residuals versus fitted values and scale–location plots (**Supplementary Figure 1**). Continuous variables are presented as mean ± standard deviation (SD). Sex differences in baseline *T*_rec_ and immune cells were examined using independent-samples t-tests, with effect sizes reported as Cohen’s *d*. Statistical significance was defined as a two-tailed *p* < 0.05.

## 3 Results

Data from 68 males and 46 females were included in the analysis. Baseline leukocyte counts did not differ between sexes (males: 6.69 ± 1.16 × 10^9^ cells·L^-^¹; females: 6.67 ± 1.40 × 10^9^ cells·L^-^¹; *p* = 0.928), nor did baseline neutrophil counts (males: 3.26 ± 0.88 × 10^9^ cells·L^-^¹; females: 3.03 ± 0.86 × 10^9^ cells·L^-^¹; *p* = 0.166). Females had a slightly higher baseline rectal temperature (*T*_rec_) than males (37.0 ± 0.18 °C *vs.* 36.8 ± 0.22 °C), but the effect size was negligible (Cohen’s *d* = 0.044). At wet-bulb temperatures (*T*_w_) of 32 °C and 33 °C, participants were exposed for 8 hours. At *T*_w_ of 34 °C and 35 °C, exposure duration ranged from 2.8 to 8 hours.

### 3.1 Relationships between rectal temperature and leukocytes and neutrophils

**Figure 1:**
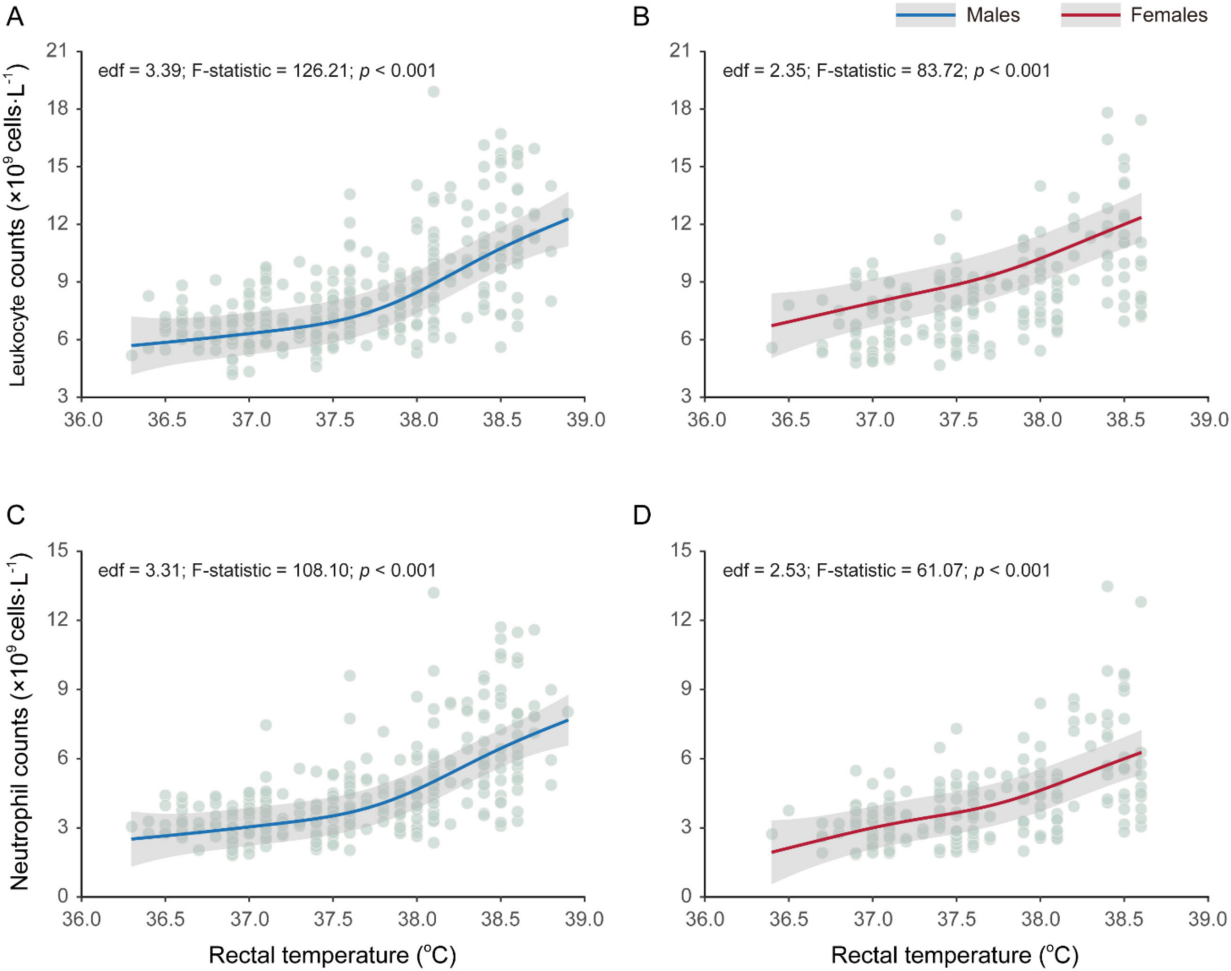
Temperature-dependent leukocyte and neutrophil changes during passive heat exposure in young adults. **(A)**, leukocyte response in males (*n* = 294 measurements); **(B)** leukocyte responses in females (*n* = 179); **(C)**, neutrophil response in males (*n* = 294); **(D)**, neutrophil responses in females (*n* = 179). Generalized additive mixed models (GAMMs) were used to characterize the population-average, non-linear relationships between *T*_rec_ and leukocyte and neutrophil counts for both sexes. For visualization, solid lines indicate population-average smooth curves, and shaded areas show 95% Wald-type confidence bands derived from fixed-effect predictions. Individual data points (grey) represent repeated measurements across participants and experimental conditions.

During prolonged passive heat exposure, both leukocyte and neutrophil counts increased progressively and nonlinearly with rising rectal temperature (*T*_rec_) in both sexes. Generalized additive mixed model (GAMM) revealed significant nonlinearity for all four combinations (edf = 2.35–3.39, all *p* < 0.001; **Figure 1**), indicating clear departures from linearity. The cell count exhibits a slow initial increase followed by a steeper rise as *T*_rec_ increased. Overall, the curves were similar between sexes for both leukocyte and neutrophil counts.

To obtain a single, interpretable does–response equation describing the association between *T*_rec_ and immune cell counts, we compared candidate parametric random-intercept mixed-effects models fitted on the log-transformed scale (**Supplementary Table 3**). Quadratic equations provided the best approximation of the population-average *T*_rec_–leukocyte and *T*_rec_*–*neutrophil relationships in both sexes and were therefore selected for reporting (**Table 1**). The reported equations correspond to the fixed-effect (population-average) component of these mixed-effects models, while between-participant heterogeneity was accounted for via random intercepts. This was reflected in an increase in explained variance from marginal *R*^2^ ( $R_{m}^{2}$) to conditional *R*^2^ ( $R_{c}^{2}$) by 0.25–0.38, indicating substantial contribution of the random intercepts to overall model fit.

**Table 1:** Population-average dose–response equations describing the association between rectal temperature (*T*_rec_) and immune cell counts.

Sex	Equation (log-transformed scale)	$R_{m}^{2}$	$R_{c}^{2}$
Males	$\ln(Y_{Leu})=0.117T_{\text{rec}}^{2}-8.540T_{\text{rec}}+157.230$	0.45	0.73
	$\ln(Y_{\text{Neu}})=0.157T_{\text{rec}}^{2}-11.377T_{\text{rec}}+207.716$	0.45	0.71
Females	$\ln(Y_{\text{Leu}})=0.060T_{\text{rec}}^{2}-4.197T_{\text{rec}}+75.203$	0.39	0.70
	$\ln(Y_{\text{Neu}})=0.069T_{\text{rec}}^{2}-4.729T_{\text{rec}}+82.114$	0.35	0.73

Equations are reported on the natural-log scale.The marginal *R*^2^ ( $R_{m}^{2}$) quantifies the variance explained by the fixed-effect dose-response equation, while the conditional *R*^2^ ( $R_{c}^{2}$) additionally includes variance explained by between-participant heterogeneity captured by the random intercept. *Y*_Leu_ and *Y*_Neu_ denote leukocyte and neutrophil counts, respectively, expressed in × 10^9^ cells·L^-1^. The rectal temperature (*T*_rec_) range included in the analysis was 36.4–38.9 °C.

### 3.2 Rectal temperature thresholds for leukocyte and neutrophil responses

**Figure 2:**
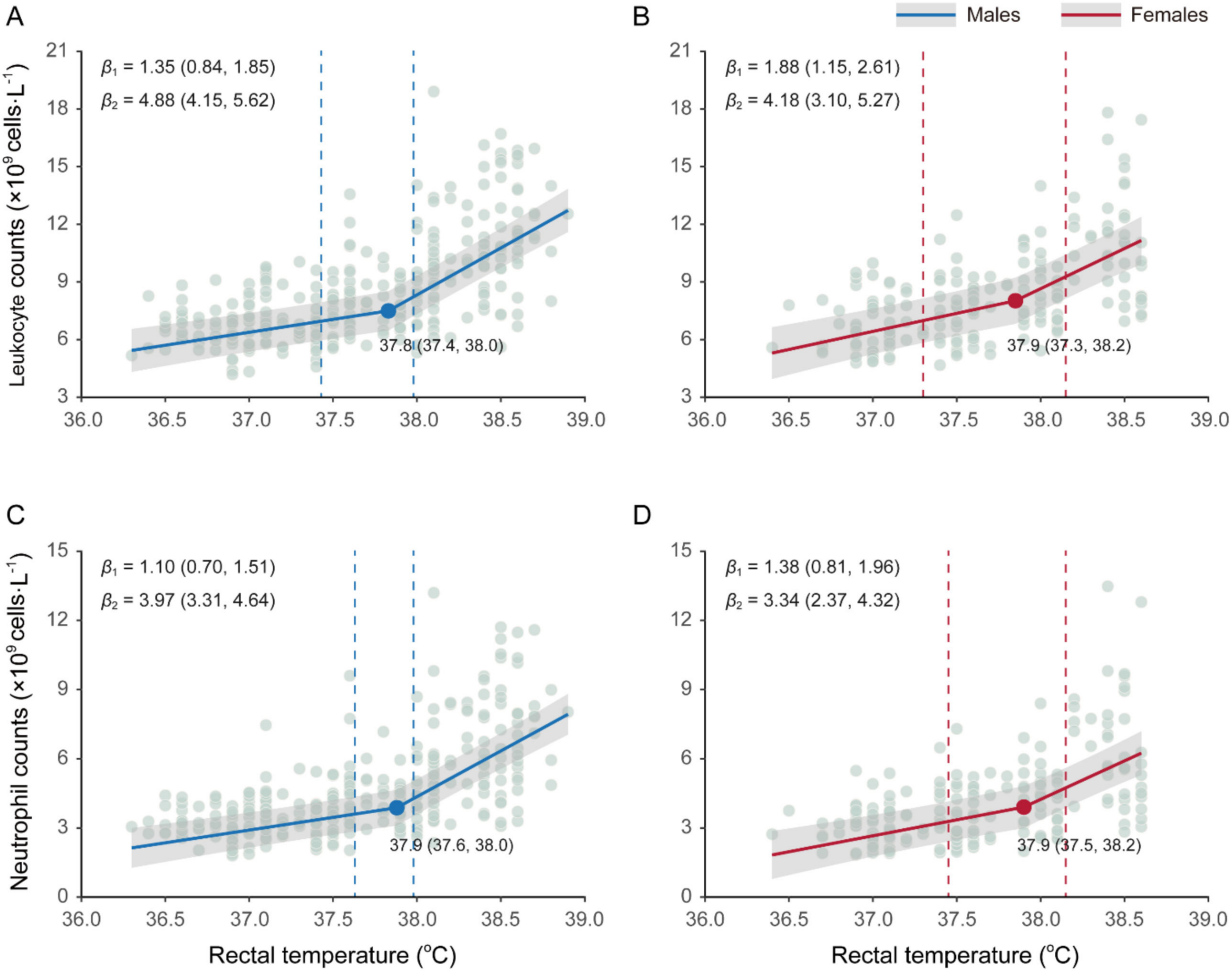
Rectal temperature (*T*_rec_) inflection points for leukocyte and neutrophil responses under prolonged stable passive heat exposure in young adults. Piecewise linear mixed-effects models were used to estimate breakpoints (solid circles) in leukocyte and neutrophil counts for males **(A, C)** and females **(B, D)**, based on the nonlinear patterns identified in **Figure 1**. All breakpoints remained significant after Bonferroni correction. Vertical dashed lines indicate 95% confidence intervals (CIs) for the breakpoints, obtained using profile likelihood (likelihood-ratio inversion) (31). Pre- and post-breakpoint slopes (*β*_1_, *β*_2_) with 95% Wald-type CIs based on fixed-effect estimates from the mixed-effects models are reported in each panel. Solid lines represent population-average fits, and shaded bands show 95% Wald-type CIs for the fixed-effect mean predictions (not individual-level prediction intervals).

Piecewise mixed-effects linear models indicated no meaningful sex difference in the estimated *T*_rec_ breakpoints for leukocyte or neutrophil counts, as evidenced by highly overlapping 95% confidence intervals (CIs). For leukocyte counts, the estimated breakpoints were 37.8 °C for males (95% CIs: 37.4–38.0 °C, *p* < 0.001) and 37.9 °C for females (95% CIs: 37.3–38.2 °C, *p* < 0.05; **Figures 2A, B**). For neutrophil counts, both sexes exhibited a significant increase (*p* < 0.05) after reaching a *T*_rec_ of 37.9 °C (males: 95% CI: 37.6–38.0 °C; females: 95% CIs: 37.5–38.2 °C; **Figure 2C, D**). In a sensitivity analysis that additionally adjusted for elapsed heat exposure time from baseline to each blood sampling point, the estimated *T*_rec_ breakpoints remained highly similar to those obtained in the original models, with overlapping confidence intervals (**Supplementary Table 4**), indicating that breakpoint identification was not materially influenced by differences in exposure duration.

Compared with the pre-breakpoint phase, post-breakpoint slopes were significantly steeper in both sexes (all *p* < 0.01), with a 3.6-fold increase in males (**Figures 2A, C**) and a 2.22–2.42-fold increase in females (**Figure 2B, D**). When *T*_rec_ was below the breakpoint, the rate of neutrophil increase with *T*_rec_ was 0.25–0.53 × 10^9^ cells·L^-1^·°C^-1^ lower than that of leukocytes; above the breakpoint, this difference increased to 0.84–0.91 × 10^9^ cells·L^-1^·°C^-1^. Before the breakpoint, rates of increase in leukocyte and neutrophil counts were modestly higher in females than in males, whereas after the breakpoint, the pattern was reversed.

## 4 Discussion

Understanding how core temperature modulates immune cell dynamics is critical in the context of increasing occupational and environmental heat stress. Here, we provided quantitative evidence linking incremental increases in core temperature to immune mobilization during prolonged passive heat exposure at wet-bulb temperatures (*T_w_*) of 32–35 °C. High-resolution rectal temperature (*T*_rec_) monitoring, combined with repeated blood sampling at 0.5 °C intervals, revealed a nonlinear increase in leukocyte and neutrophil counts. Notably, when *T*_rec_ approached approximately 37.8–37.9 °C, the rate of immune cell increase rose sharply in both sexes (**Figure 2**), revealing a clear immunological inflection point. These findings define a core temperature–dependent threshold for the mobilization of circulating innate immune cells, which coincides with established limits of thermoregulatory compensation in humans.

### 4.1 Core temperature thresholds and sex-specific mobilization of circulating innate immune cells

The 38 °C occupational core temperature limit recommended by the World Health Organization (WHO) corresponds to a workload of approximately 490 W (13). Average core temperatures above 38 °C in worker populations are most likely to occur at 500 W or higher (32). In our study, we identified a consistent core temperature breakpoint of 37.8–37.9 °C (with overlapping 95% CI across models) during light physical activity (~1.2–1.5 METs), at which leukocyte and neutrophil counts accelerated significantly in both sexes. This threshold was independent of cell type and showed no meaningful sex difference in location (**Figure 2**), suggesting a unified physiological trigger for enhanced innate immune mobilization during passive heat stress. Kappel et al. (33) reported that *T*_rec_=38.0 °C increased male immune cell counts using a 2-hour water bath protocol, with median leukocyte counts of 5.25 × 10^9^ cells·L^-1^ and neutrophil counts of 2.45 × 10^9^ cells·L^-1^. In the present study, at *T*_rec_ ≥ 38 °C, most post-breakpoint leukocyte counts exceed normal reference ranges (males: 10.58 ± 2.84 × 10^9^ cells·L^-1^; females: 10 ± 2.75× 10^9^ cells·L^-1^), as did neutrophil counts (males: 6.23 ± 2.34 × 10^9^ cells·L^-1^; females: 5.50 ± 2.39 × 10^9^ cells·L^-1^) (30). These findings provide direct immunological validation for the longstanding 38 °C threshold.

The patterns of leukocyte and neutrophil mobilization exhibit sex-specific differences. Below the *T*_rec_ breakpoint, females exhibited a steeper increase in both leukocyte and neutrophil counts (**Figures 2B, D**), indicating earlier mobilization. Above the breakpoint, males displayed a 16.7–18.9% greater slope, particularly for leukocytes (**Figures 2A, C**), indicating more pronounced immune mobilization at higher core temperatures. These phenomena likely reflect integrated effects of hormonal modulation, thermoregulatory control, and hematopoietic capacity. Testosterone promotes pro-inflammatory signalling via androgen receptor-mediated pathways (34), whereas estrogen suppresses nuclear factor kappa-B (NF-κB) activation and enhances anti-inflammatory cytokine production (35), which may explain the earlier leukocyte mobilization in females followed by a plateau due to anti-inflammatory feedback. In addition, males possess a larger leukocyte reserve pool in the bone marrow (36), potentially enabling delayed but more robust mobilization once a critical core temperature threshold is surpassed. Other factors, including sex differences in body composition, heat storage capacity, and cardiovascular strain, likely also contribute to these response patterns. Supporting this interpretation, heart rate increased from pre- to post-exposure across all heat exposure conditions (**Supplementary Table 1**), consistent with progressive cardiovascular strain during prolonged heat exposure and reflecting the increasing physiological burden accompanying rising core temperature.

### 4.2 Mechanisms underlying rapid leukocyte mobilization

The rapid, core temperature-dependent rise in circulating leukocytes and neutrophils can occur through multiple pathways that do not require concurrent cytokine elevations. Acute stressors elicit leukocyte demargination via catecholamine- and glucocorticoid-dependent mechanisms, leading to marginated cells detaching from the endothelium and entering the circulation (37). These processes can occur within minutes and are facilitated by changes in cell deformability and vascular shear forces. On a slightly longer timescale, bone marrow release may further augment circulating cell numbers. Importantly, these mechanisms precede, or operate independently of, the slower, cytokine-driven inflammatory cascades.

This distinction has both mechanistic and applied significance. Rapid leukocyte mobilization likely represents an adaptive, transient redistribution of immune cells to maintain host defense during acute heat stress. However, sustained activation at elevated core temperatures could amplify systemic inflammation, with potential implications for heat-related illness risk. Clinically, early immune activation may lower the threshold for inflammatory sequelae such as endothelial dysfunction, coagulopathy, or systemic inflammatory response, thereby increasing vulnerability during prolonged heat exposure.

### 4.3 Integrating molecular mediators and thermoregulatory control

Although molecular mediators were not measured in the present study, previous studies have documented increases in interleukin-6 (IL-6), tumor necrosis factor-alpha (TNF-α), and granulocyte colony-stimulating factor (G-CSF) with increasing thermal strain (38). Central thermoregulatory circuits and the hypothalamic-pituitary-adrenal (HPA) axis likely modulate these responses through sympathetic activation and glucocorticoid release (39, 40). Moreover, the transient receptor potential vanilloid 1 (TRPV1) channel, which detects both peripheral and core heating, may act as an upstream molecular sensor linking thermal inputs to immune pathways (41). Future studies incorporating cytokine profiling, cell surface activation markers (e.g., CD62L shedding), and neuroendocrine measures will be valuable for delineating the relative contribution of demargination versus *de novo* immune activation.

### 4.4 Practical and translational implications

The estimated core temperature breakpoint for accelerated leukocyte and neutrophil responses occurred at approximately 38 °C, suggesting a potential physiological reference point relevant to current occupational exposure frameworks. Our findings support the potential values of integrating immune-informed monitoring into heat strain guidelines, complementing recent individualized approaches, such as those in the 2024 ACGIH update (42), which emphasize physiological monitoring (e.g., limiting core temperature rise to 1 °C above pre-job values as a dynamic indicator of heat strain rather than relying on a fixed core temperature threshold). Incorporating both physiological and environmental metrics may enable more flexible and responsive management of heat exposure. While the present findings do not establish a definitive clinical threshold, they suggest that elevations in core temperature approaching ~38 °C are associated with measurable changes in circulating immune markers, which may reflect early physiological strain. More broadly, these temperature-associated increases in circulating leukocytes and neutrophils may represent an early systemic stress response that precedes overt clinical heat illness. If sustained or repeatedly triggered, such changes could contribute to a pro-inflammatory milieu linked to endothelial dysfunction, coagulation imbalance, or processes implicated in heat-related illness (22, 43), and may have practical relevance for identifying early physiological strain in individuals exposed to extreme heat before the onset of overt clinical symptoms.

### 4.5 Limitations and future directions

Several limitations should be acknowledged. First, while cytokine and hormonal measurements were not included in this study due to budgetary constraints, their inclusion would have allowed for a more direct investigation into the mechanistic pathways underlying the observed leukocytosis. Second, the study focused on healthy young adults, and results may not generalize to older individuals or those with comorbidities, who may exhibit different thermal and immune response profiles. The circulating immune cell counts are known to exhibit diurnal variation (44). Although all trials were initiated at 09:00, time-of-day effects over the prolonged exposure period cannot be fully excluded. In addition, while sensitivity analyses indicated that breakpoint estimates were robust to elapsed exposure time, the independent contribution of exposure duration to immune mobilization cannot be fully excluded. Third, only passive exposure with light activity was tested in the present study. Many individuals exposed to high temperatures perform moderate-to high- intensity physical work, which increases metabolic heat production and may accelerate the rise in core temperature. Under such conditions, immune cell mobilization may occur earlier or be more pronounced than observed here. Finally, blood sampling at 0.5 °C increments may limit resolution around the identified breakpoint, and future studies should consider more frequent sampling near this transition to better characterize the onset of immune mobilization. Future research should integrate immune, endocrine, and thermoregulatory markers in real time to more comprehensively map the temporal sequence of these responses. Longitudinal studies are also needed to evaluate whether repeated or chronic heat exposure leads to immune adaptation or maladaptation.

## 5 Conclusions

Collectively, we identified a clear nonlinear infection in leukocyte and neutrophil dynamics during prolonged heat exposure, with accelerated mobilization occurring at core temperatures of approximately 38.0 °C, which is comparable to the commonly cited threshold for limiting excessive heat strain. This rapid immunological mobilization exhibits distinct sex-dependent patterns, with moderated post-threshold acceleration in females and steeper increases in males and may serve as an early biological indicator of systemic strain beyond thermoregulatory limits. These findings extend the physiological rationale for current occupational heat exposure thresholds and underscore the value of incorporating immune response markers alongside traditional cardiovascular and thermoregulatory measures to refine safe exposure limits and guide individualized monitoring strategies in high-risk settings.

## Data Availability

The original contributions presented in the study are included in the article/**Supplementary Material**. Further inquiries can be directed to the corresponding author.
