# Supplementary material for: Core temperature-dependent leukocyte and neutrophil responses during prolonged heat exposure
Source: Front Immunol. 2026 Apr 22;17:1798608. doi: 10.3389/fimmu.2026.1798608 (PMC13143634; doi:10.3389/fimmu.2026.1798608)
Supplement: Supplementary file 1 [file DataSheet1.docx]

**Supplemental Material**

**Core Temperature Dependent Leukocyte and Neutrophil Responses During Prolonged Heat Exposure**

**By**

Yi Xu, Haojian Wang, Fèlix Faming Wang^*^

This supplementary file contains one figure and four tables.

**Supplementary Fig. 1 | Residual diagnostics for the piecewise linear mixed-effects models, including Pearson residuals versus predicted leukocyte and neutrophil counts (A–D) and scale–location plots (E–H).** The grey band represents a locally smoothed trend with its 95% confidence interval. The plots suggest a moderate increase in variance at higher predicted response values, consistent with the greater dispersion observed in the raw data, but do not indicate clear systematic deviations from model assumptions across the range of core temperatures.

**
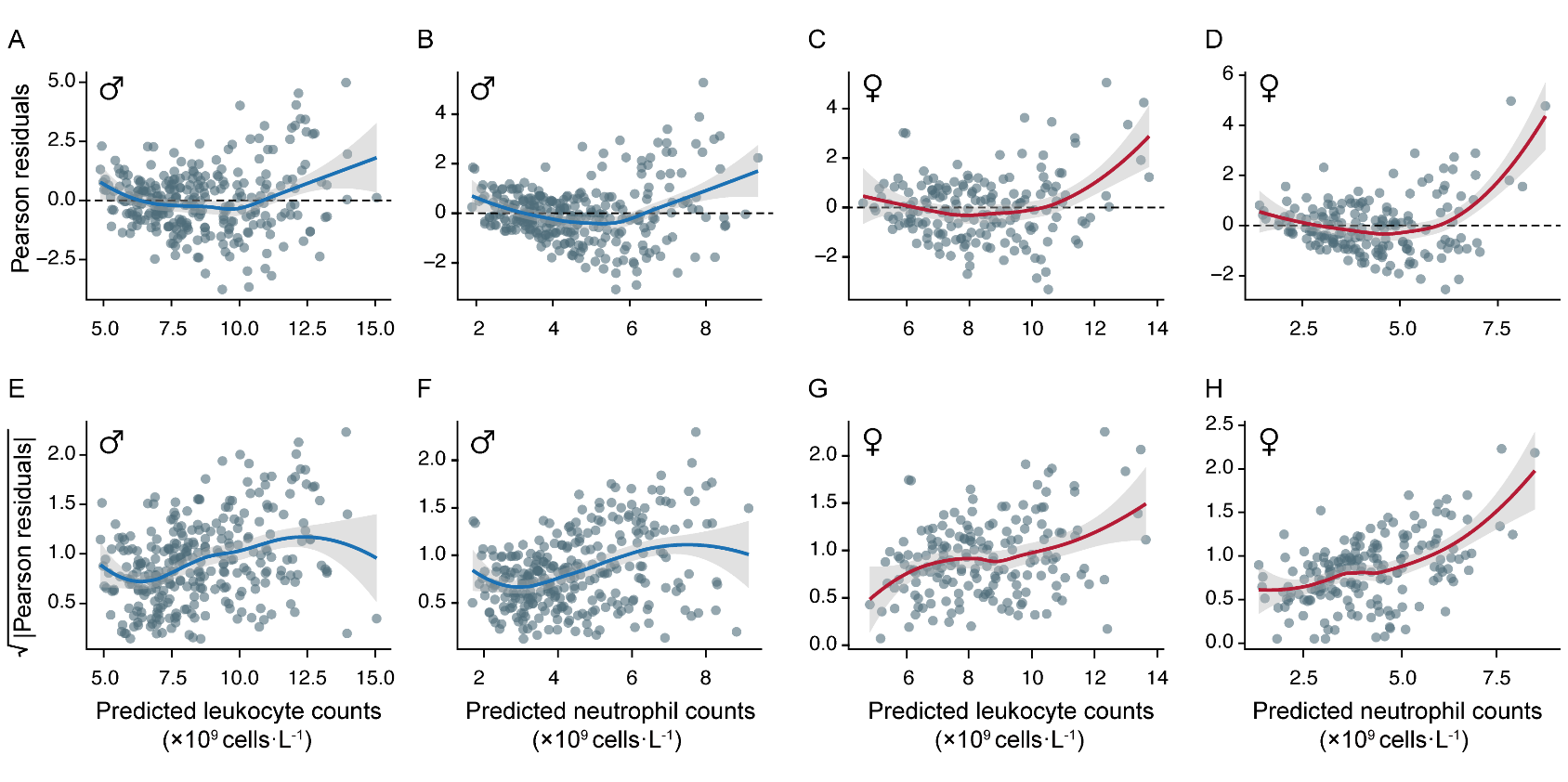
**

**Supplementary Table S1 | Baseline anthropometric and physiological characteristics for both sexes across four heat exposure scenarios at wet-bulb temperatures (*T*_w_) of 32 °C, 33 °C, 34 °C, and 35 °C (exposure time: 2.8–8.0 h).** Values are presented as mean ± SD for participants measured within each experimental condition. BMI, body mass index; BSA, body surface area; *T_db_*, dry-bulb temperature; RH, relative humidity; *T_rec_*, rectal temperature; Pre-/Post-HR, heart rate before and after exposure; Pre-/Post-USG, urine specific gravity before and after heat exposure; SR, sweat rate; WI, water intake per hour during exposure.

| ***T*_w_** | ***T*_w_ = 32°C** | | | | | | ***T*_w_ = 33°C** | | | | | |
| --- | --- | --- | --- | --- | --- | --- | --- | --- | --- | --- | --- | --- |
| ***T_db_* & RH** | **46.0±0.2°C & 34.8±0.6%** | | **39.9±0.5 °C & 57.7±2%** | | **36.1±0.1 °C & 74.1±1.2%** | | **37.3±0.1 °C & 75.3±0.5%** | | **42.3±0.1 °C & 51.5±0.6%** | | **47.1±0.2 °C & 35.2±0.7%** | |
| Sex | Males | Females | Males | Females | Males | Females | Males | Females | Males | Females | Males | Females |
| Number | 20 | 16 | 68 | 58 | 20 | 16 | 20 | 16 | 20 | 16 | 20 | 16 |
| Age (yr) | 24.9 ± 2.3 | 23.0 ± 2.2 | 24.7 ± 1.5 | 23.1 ± 2.2 | 24.6 ± 0.7 | 22.9 ± 1.9 | 24.6 ± 2.3 | 23.4 ± 1.9 | 24.3 ± 2.3 | 23.4 ± 1.9 | 24.3 ± 2.3 | 23.5 ± 1.8 |
| BMI (kg/m^2^) | 22.1 ± 1.8 | 21.1 ± 2.3 | 21.9 ± 1.3 | 20.1 ± 1.9 | 22.8 ± 1.4 | 20.4 ± 1.8 | 22.2 ± 2.1 | 20.8 ± 2.0 | 22.4 ± 1.5 | 20.8 ± 2.0 | 22.2 ± 1.7 | 20.5 ± 2.1 |
| BSA (m^2^) | 1.87 ± 0.10 | 1.59 ± 0.10 | 2.0 ± 0.12 | 1.65 ± 0.11 | 1.90 ± 0.14 | 1.59 ± 0.09 | 1.82 ± 0.15 | 1.53 ± 0.11 | 1.83 ± 0.10 | 1.53 ± 0.11 | 1.84 ± 0.10 | 1.52 ± 0.11 |
| Pre-HR (pbm) | 75 ± 4 | 72 ± 5 | 73 ± 4 | 73 ± 4 | 76 ± 6 | 78 ± 6 | 77 ± 6 | 74 ± 4 | 74 ± 5 | 73 ± 6 | 74 ± 4 | 75 ± 4 |
| Post-HR (pbm) | 125 ± 9 | 115 ± 11 | 110 ± 15 | 110 ± 14 | 104 ± 14 | 108 ± 13 | 112 ± 10 | 112 ± 11 | 113 ± 9 | 116 ± 15 | 117 ± 10 | 120 ± 13 |
| Pre-USG | 1.013 ± 0.005 | 1.005 ± 0.004 | 1.016 ± 0.005 | 1.011 ± 0.001 | 1.008 ± 0.005 | 1.008 ± 0.004 | 1.011 ± 0.004 | 1.014 ± 0.003 | 1.010 ± 0.004 | 1.009 ± 0.004 | 1.012 ± 0.003 | 1.011 ± 0.003 |
| Post-USG | 1.016 ± 0.008 | 1.006 ± 0.003 | 1.014 ± 0.008 | 1.008 ± 0.001 | 1.007 ± 0.009 | 1.005 ± 0.006 | 1.015 ± 0.005 | 1.012 ± 0.004 | 1.016 ± 0.005 | 1.014 ±0.004 | 1.020 ± 0.005 | 1.015 ±0.005 |
| SR (g/h) | 195 ± 44 | 368 ± 54 | 278 ± 79 | 223 ± 53 | 310 ± 90 | 138 ± 74 | 231 ± 68 | 202 ± 52 | 296 ± 46 | 236 ± 62 | 346 ± 57 | 282 ± 47 |
| WI (ml/h) | 375 ± 70 | 454 ± 12 | 368 ± 25 | 386 ± 38 | 353 ± 75 | 389 ± 49 | 272 ± 17 | 254 ± 19 | 281 ± 10 | 278 ± 10 | 302 ± 13 | 287 ± 15 |
| ***T*_w_** | ***T_w_* = 34°C** | | | | | | ***T_w_* = 35°C** | | | | | |
| ***T_db_* & RH** | **38.3±0.1 °C & 75.2±0.5%** | | **43.3±0.2 °C & 52.1±0.5%** | | **48.1±0.2 °C & 35.9±0.6%** | | **40.6±0.3 °C & 69.5±1.4%** | | **45.3±0.4 °C & 50.3±1.3%** | | **50.2±0.6 °C & 35.0±1.3%** | |
| Sex | Males | Females | Males | Females | Males | Females | Males | Females | Males | Females | Males | Females |
| Number | 20 | 16 | 20 | 16 | 20 | 16 | 20 | 16 | 20 | 16 | 20 | 16 |
| Age (yr) | 24.3 ± 1.8 | 23.4 ± 1.9 | 24.3 ± 1.8 | 23.4 ± 1.8 | 24.3 ± 1.8 | 23.4 ± 1.8 | 24.9 ± 4.0 | 23.0 ± 2.2 | 24.6 ± 4.0 | 23.0 ± 2.2 | 24.3 ± 1.8 | 22.9 ± 2.5 |
| BMI (kg/m^2^) | 21.9 ± 1.3 | 21.4 ± 2.2 | 21.9± 1.3 | 21.2 ± 2.2 | 22.7 ± 1.2 | 21.2 ± 2.2 | 22.1 ± 1.8 | 20.9 ± 1.4 | 22.9 ± 0.8 | 20.9 ± 1.4 | 21.6 ± 2.3 | 20.7 ± 1.5 |
| BSA (m^2^) | 1.83 ± 0.08 | 1.57 ± 0.11 | 1.83 ± 0.08 | 1.57 ± 0.11 | 1.83 ± 0.08 | 1.57 ± 0.11 | 1.85 ± 0.09 | 1.64± 0.08 | 1.86 ± 0.09 | 1.64 ± 0.08 | 1.86 ± 0.10 | 1.63 ± 0.09 |
| Pre-HR (pbm) | 75 ± 5 | 76 ± 5 | 76 ± 6 | 74 ± 6 | 76 ± 4 | 75 ± 5 | 75 ± 6 | 71 ± 4 | 74 ± 6 | 72 ± 5 | 76 ± 4 | 73 ± 4 |
| Post-HR (pbm) | 114 ± 10 | 119 ± 13 | 120 ± 13 | 118 ± 11 | 121 ± 15 | 124 ± 15 | 120 ± 11 | 129 ± 9 | 120 ± 12 | 128 ± 12 | 122 ± 10 | 130 ± 9 |
| Pre-USG | 1.014 ± 0.004 | 1.011 ± 0.003 | 1.013 ± 0.003 | 1.013 ± 0.004 | 1.013 ± 0.004 | 1.013 ± 0.005 | 1.017 ± 0.002 | 1.015 ± 0.002 | 1.015 ± 0.003 | 1.014 ± 0.002 | 1.016 ± 0.003 | 1.014 ± 0.005 |
| Post-USG | 1.009 ± 0.004 | 1.007 ± 0.004 | 1.010 ± 0.005 | 1.009 ± 0.007 | 1.011 ± 0.007 | 1.011 ± 0.005 | 1.008 ± 0.003 | 1.008 ± 0.003 | 1.010 ± 0.003 | 1.008 ± 0.002 | 1.012 ± 0.004 | 1.004 ± 0.003 |
| SR (g/h) | 316 ± 79 | 209 ± 68 | 365 ± 75 | 256 ± 81 | 368 ± 132 | 357 ± 90 | 437 ± 137 | 428 ± 165 | 462 ± 126 | 410 ± 87 | 435 ± 131 | 455 ± 167 |
| WI (ml/h) | 472 ± 90 | 524 ± 67 | 479 ± 91 | 509 ± 73 | 520 ± 100 | 551 ± 74 | 587 ± 97 | 586 ± 83 | 626 ± 154 | 564 ± 93 | 557 ± 195 | 604 ± 100 |

**Supplementary Table S2 | Comparison of dehydration rate (DR, %) between sexes across the heat exposure conditions.** Negative DR values indicate net fluid gain (fluid intake exceeding total fluid losses), whereas positive values indicate net dehydration. No participant exhibited a DR exceeding 1%, indicating that hydration status was well maintained during the trials. *T_db_*, dry-bulb temperature; RH, relative humidity.

| ***T_w_* (°C)** | ***T_db_* (°C)** | **RH (%)** | **Dehydration Rate (%)** | |
| --- | --- | --- | --- | --- |
|  |  |  | **Males** | **Females** |
| 32 | 46.0 | 34.8 | 0.54 ± 0.17 | 0.63 ± 0.16 |
|  | 39.9 | 57.7 | -0.30 ± 0.67 | -0.49 ± 0.51 |
|  | 36.1 | 74.1 | 0.35 ± 0.26 | 0.43 ± 0.32 |
| 33 | 37.3 | 75.3 | 0.27 ± 0.56 | -0.17 ± 0.75 |
|  | 42.3 | 51.5 | 0.76 ± 0.15 | 0.59 ± 0.40 |
|  | 47.1 | 35.2 | 0.55 ± 0.37 | 0.69 ± 0.35 |
| 34 | 38.3 | 75.2 | -0.52 ± 0.54 | -0.59 ± 0.46 |
|  | 43.3 | 52.1 | -0.06 ± 0.03 | -0.67 ± 0.58 |
|  | 48.1 | 35.9 | -0.05 ± 0.29 | -0.50 ± 0.53 |
| 35 | 40.6 | 69.5 | -0.47 ± 0.35 | -0.56 ± 1.06 |
|  | 45.3 | 50.3 | -0.60 ± 0.59 | -0.50 ± 0.31 |
|  | 50.2 | 35.0 | -0.25 ± 0.62 | -0.44 ± 0.41 |

The dehydration rate (*DR*) was calculated from Equation (1), expressed as a percentage, which quantifies the degree of dehydration resulting from body water loss during the experiment.

$DR=\frac{SP+UL-FI}{IBW}\cdot100\%$ (1)

where, *SP* (sweat production) is the total volume of sweat secreted during the observation period (mL); *UL* (urine loss) is the total volume of urine excreted (mL); *FI* (fluid intake) includes all sources of water uptake (mL); *IBW* (initial body weight) is the participant’s body mass before exposure (kg), used to normalize water loss and enable consistent comparisons of relative dehydration.

**Supplementary Table S3 | Model comparison for parametric dose–response relationships between rectal temperature (*T*_rec_) and immune cell counts.** Leukocyte and neutrophil counts (×10⁹/L) were fitted on the natural-log scale. Linear, quadratic, and power random-intercept mixed-effects models were fitted separately for males and females. Model performance was assessed using Akaike information criterion (AIC), Bayesian information criterion (BIC), and the marginal coefficient of determination ($\text{R}_{\text{m}}^{\text{2}}$), which reflects the variance explained by fixed effects; lower AIC and BIC indicate better model fit. Model comparisons were based on the valid analytical dataset used in the present study, comprising 294 observations from 68 males and 179 observations from 46 females.

| **Models** | **Males** | | | **Females** | | |
| --- | --- | --- | --- | --- | --- | --- |
|  | **AIC** | **BIC** | ***R*_m_^2^** | **AIC** | **BIC** | $\text{R}_{\text{m}}^{\text{2}}$ |
|  | Leukocyte counts transformed by natural logarithm, expressed in × 10^9^/L | | | | | |
| Linear regression | -128.92 | -114.19 | 0.422 | -66.12 | -53.37 | 0.385 |
| Quadratic regression | -150.66 | -132.24 | 0.445 | -66.37 | -50.43 | 0.390 |
| Power regression | -127.38 | -112.65 | 0.420 | -65.80 | -53.05 | 0.385 |
|  | Neutrophil counts transformed by natural logarithm, expressed in × 10^9^/L | | | | | |
| Linear regression | 79.58 | 94.31 | 0.423 | 64.56 | 77.30 | 0.348 |
| Quadratic regression | 61.44 | 79.86 | 0.447 | 65.05 | 80.99 | 0.351 |
| Power regression | 80.97 | 95.70 | 0.422 | 64.82 | 77.57 | 0.347 |

**Supplementary Table S4 | Sensitivity analysis examining the effects of rectal temperature (*T*_rec_) and elapsed heat exposure time on circulating immune cell responses.** Breakpoint estimates and 95% confidence intervals (CIs) from the original and time-adjusted piecewise mixed-effects models are presented. Breakpoint estimates were highly similar between models, with overlapping confidence intervals, indicating that the identification of temperature-associated changes in circulating immune cell responses was not materially influenced by adjustment for elapsed time. *p*, significance.

| **Sex** | **Outcome** | **Model** | ***T*_rec_ breakpoint** | **Time effect (*p*)** |
| --- | --- | --- | --- | --- |
| Males | Leukocytes | Original | 37.8 (37.4, 38.0) |  |
|  |  | Time-adjusted | 37.9 (37.7, 38.0) | < 0.0001 |
| Males | Neutrophils | Original | 37.9 (37.6, 38.0) |  |
|  |  | Time-adjusted | 37.9 (37.7, 38.0) | < 0.0001 |
| Females | Leukocytes | Original | 37.9 (37.3, 38.2) |  |
|  |  | Time-adjusted | 38.1 (37.6, 38.2) | 0.0002 |
| Females | Neutrophils | Original | 37.9 (37.5, 38.2) |  |
|  |  | Time-adjusted | 38.1 (37.8, 38.2) | < 0.0001 |
